# Characteristics and treatment response of self-identified problematic Internet users in a behavioral addiction outpatient clinic

**DOI:** 10.1556/JBA.3.2014.008

**Published:** 2014-02-03

**Authors:** Gabriel Thorens, Sophia Achab, Joël Billieux, Yasser Khazaal, Riaz Khan, Edward Pivin, Vishal Gupta, Daniel Zullino

**Affiliations:** ^1^University Hospital of Geneva, Geneva, Switzerland; ^2^Laboratory for Experimental Psychopathology, Psychological Science Research Institute, Catholic University of Louvain, Louvain-la-Neuve, Belgium; ^3^Lausanne University Hospital, Lausanne, Switzerland; ^4^Department of Psychiatry, NYU School of Medicine, New York, USA

**Keywords:** Internet, problematic, usage, addiction, gaming

## Abstract

*Aims:* Controversies remain about the validity of the diagnosis of problematic Internet use. This might be due in part to the lack of longitudinal naturalistic studies that have followed a cohort of patients who self-identify as having Internet-related problems. *Methods:* This retrospective study included 57 patients who consulted the Geneva Addiction Outpatient Clinic from January 1, 2007, to January 1, 2010. Patients underwent an initial clinical psychiatric evaluation that included collection of data on socio-demographics, method of referral, specific Internet usage, psychiatric diagnosis, and Internet Addiction Test (IAT) and Clinical Global Impression Scale (CGI) scores. Treatment consisted of individual psychotherapeutic sessions. *Results:* Of these patients, 98% were male and 37% were 18 years or younger. Most patients were online gamers (46% playing massively multiplayer online role-playing games). The mean IAT score was 52.9 (range 20–90). Sixty-eight percent of patients had a co-morbid psychiatric diagnosis, with social phobia being the most prevalent (17.8%). Patients who remained in treatment (dropout rate 24%) showed an overall improvement of symptoms: 38.6% showed significant or average improvement on their CGI score, 26.3% showed minimal improvement, and 14% showed no change. *Conclusions:* Our results support the hypothesis that there are specific types of Internet use, with online gaming mainly affecting young male patients. As Internet addiction is not yet an official diagnosis, better instruments are needed to screen patients and to avoid false-negative and false-positive diagnoses. Successful care should integrate the treatment of co-morbid symptoms and involve families and relatives in the therapeutic process.

## Introduction

Naturalistic studies have rarely examined a cohort of patients who self-identify as having Internet-related problems in dedicated clinics. Moreover, few case studies describe the evolution of patients who have mainly been treated with cognitive behavioral therapy (Allison, von Wahlde, Shockley & Gabbard, 2006; King, Delfabbro, Griffiths & Gradisar, 2012; Thorens, Khazaal & Zullino, 2012).

This paucity of clinical research might be because controversies remain regarding the diagnosis of problematic Internet use (PIU), despite a growing body of literature on the potential problematic mental health issues caused by inappropriate Internet use. Multiple terms and definitions have been proposed, as inappropriate Internet use is sometimes considered to be a behavioral addiction, similar to gambling, in terms of the conditioning rewards mechanism and clinical consequences (Demetrovics et al., 2011; Griffiths & Demetrovics, 2012).

Han et al. (2009) rejected the existence of a formal diagnosis of Internet addiction, postulating that PIU is a symptom of already well-defined clinical entities such as depression, social phobia, or attention deficit hyperactivity disorder (ADHD). Tao et al. (2010), on the other hand, defined the diagnostic criteria for Internet addiction. Such controversy may arise from the types of studies published to date, most of which are observational or cross-sectional surveys (Van Rooij, Schoenmakers, Vermulst, Van den Eijnden & Van de Mheen, 2011) of non-treatment-seeking individuals (generally high school or college students). A meta-synthesis exposed the issue raised by the lack of defined diagnostic criteria and selection bias (Byun et al., 2009). The actual definition of Internet addiction does not take into account that only certain Internet-specific usage is likely to pose a risk of addiction, such as video games or sexual content (Griffiths, 2012; Kuss & Griffiths, 2012; Van Rooij et al., 2011).

Clinical trials that focus on treatment and evaluation of Internet addiction are described as having major limitations. A recent review of eight treatment studies shows poor evaluation methods, outcomes, and patient selection (King, Delfabbro, Griffiths & Gradisar, 2011). In this review, the researchers identified eight studies: six were conducted in Asia (South Korea, China, and Hong Kong), two in the United States, and none in Europe. Among these eight studies, only one was a randomized control trial. One of the main concerns was that the studies lacked a description of the inclusion and exclusion criteria and of the method of recruitment. Another concern was that patients included in the clinical group may have exhibited very different types of psychological functioning, primary versus secondary disorders, and specific usage (gaming, chatting, sexual content, etc.).

Our study examined a cohort of patients who self-identified as having Internet-related problems. This presented an opportunity to describe the treatment and evolution of these patients, as well as to characterize them in terms of demographics, co-morbidities, and response to treatment.

## Methods

### Procedure

In 2007, the Addiction Division of Geneva University Hospital opened an outpatient clinic dedicated to the treatment of behavioral addictions (PIU, pathological gambling, and compulsive buying). This facility is affiliated with the Geneva University public hospitals and is accessible to every patient without restriction. Patients have the option to walk in or to be referred by anyone (family or other health professionals). The current retrospective study examined the clinical charts of all patients with PIU as the main motive for consultation between January 1, 2007, and January 1, 2010.

After an initial clinical psychiatric evaluation of the patients by an addiction specialist (MD), treatment consisted of individual psychotherapy sessions. Psychotherapy included standard addiction cognitive behavioral therapy and motivational interview components adapted to PIU. Patients with co-morbid psychiatric disorders were screened and treated for concomitant disorders. Patients with a psychiatric diagnosis and no addiction symptoms were screened and referred to general psychiatric treatment facilities.

### Measures

At the first session, the following data were collected: socio-demographics (gender, age), method of referral, previous psychiatric treatments, specific Internet usage (gaming, surfing, etc.), psychiatric diagnosis (based on a standard semi-structured interview on the first encounter with participants), co-morbid addictions, and scores on two scales (described below). We also collected data on length of treatment, number of sessions, dropout rate, and psychiatric medication use for co-morbid diagnosis.

### Scales

Patients completed the French version of the Internet Addiction Test (IAT) (Khazaal et al., 2008), an adaptation of the IAT developed by Young (1998). It consists of the 20 original items. All items are scored on a Likert scale (never, rarely, occasionally, often, always) corresponding to scores from 1 to 5. The range of the scale is from 20 (answering “never” to all questions) to 100. We chose 70 as the cut-off for pathological Internet use, on the basis of a 2013 review of Internet addiction assessment tools by Lortie and Guitton (2013). The authors of this review commented on the heterogeneous cut-off values in studies that used the IAT and recommended choosing strict selection criteria to avoid false-positive results.

Patients were also evaluated with the Clinical Global Impression Scale (CGI) (Busner & Targum, 2007), which is a widely used and validated clinical tool to assess clinical severity and evolution.

### Participants

The sample consisted of 57 participants who are all included in the descriptive statistical analysis. Missing data have been taken into account by adjusting the number of total answers (N).

### Ethics

The ethical committee of Geneva University Hospital approved this study.

## Results

The mean age of patients was 24 years (range 13–67). All were male except one. The mean IAT score for our sample was 52.9 (range 20–90). Results are presented in Tables 1 and 2 (the variation in sample size is due to missing data).

**Table 1.** Motives for referral and psychiatric diagnosis (*N* = 56)

Measure	Frequency	Percentage (%)
Referred by		
Family	31	55.3
Themselves	12	21.4
Social care	7	12.6
Caregiver	6	10.7
Treatment-seeking motives		
MMORPGs	26	46.4
Multipurpose	10	17.8
Sexual content	7	12.5
Chatting	6	10.7
First-person shooter games	3	5.4
Other games	3	5.4
Surfing	1	1.8
Previous psychiatric care		
None	34	60.7
Private practice	15	26.8
Institution	7	12.5
Current psychiatric diagnosis		
None	17	30.4
Social phobia	10	17.8
Psychosis	6	10.7
Addiction	6	10.7
Anxiety disorder	5	8.9
ADHD	4	7.1
Mood disorder	3	5.4
Personality disorder	3	5.4
Bipolar disorder	1	1.8
Mental retardation	1	1.8

*Note:* MMORPGs = massively multiplayer online role-playing games; ADHD = attention deficit hyperactivity disorder.

Table 1 shows the method of referral, treatment-seeking motives, prior psychiatric care, and current psychiatric diagnoses. Most patients were referred by family members, and the main motive for consultation was gaming (massively multiplayer online role-playing games [MMORPGs] was the main overall motive for consultations). Most patients (60%) had never had treatment before. The results of the IAT showed that, with a cutoff of 70 points used as the limit to indicate having PIU, only 18.7% (9) of the 48 patients with an available IAT score were above that score. The co-morbidity diagnosis figures show that 67.9% of patients have a co-morbid psychiatric diagnosis. Social phobia was the most prevalent of these (17.8%).

Table 2 shows the CGI at baseline and evolution, as well as the dropout rate. The severity of clinical symptoms was generally in the lower range (8.7% were evaluated as having no symptoms) and the overall evolution was favorable (did not worsen during treatment). The mean length of treatment in our sample was 19.1 weeks (range 0–100) and six sessions (range 1–40). The dropout rate was 24.6%. Regarding concomitant substance use, most of the sample, 70.4%, did not use drugs (38 of 54); 11.1% smoked tobacco (6 of 54); 9.3% used THC (5 of 54); 3.7% used alcohol (2 of 54); 3.7% used NMDA (2 of 54); and 1.8% used multiple drugs (1 of 54).

**Table 2.** CGI baseline, evolution, and dropout rate (*N* = 57)

Measure	Frequency	Percentage (%)
CGI baseline		
Normal, not at all ill	5	8.7
Borderline mentally ill	5	8.7
Mildly ill	9	15.7
Moderately ill	14	24.5
Markedly ill	17	29.8
Severely ill	7	13
Among the most extremely ill	0	0
CGI evolution		
Very much improved	7	12.3
Much improved	15	26.3
Minimally improved	15	26.3
No change	8	14
Minimally worse	0	0
Much worse	0	0
Very much worse	0	0
Dropout rate	14	24.6

*Note:* CGI = Clinical Global Impression Scale.

We found no statistical differences (*t-*test) in the IAT scores of patients with a co-morbid psychiatric diagnosis compared with patients without a psychiatric diagnosis.

## Discussion

This retrospective study shows the baseline characteristics and the evolution of a cohort of self-identified patients with Internet-related problems. Most of the patients were young males, which is consistent with epidemiological studies that show a predominance of male adolescents among those that have PIU. Our cohort shows that most of these patients are online gamers playing MMOPRGs. This finding emphasizes the latest results that describe the population of those with PIU and confirms that online gaming (and more specifically MMORPGs) might be one of the most addictive and potentially harmful forms of Internet use.

A minority of patients consulted the clinic for the use of sexual content on the Internet. This specific usage also brings up the question of the overlap of diagnosis between sexual addiction and sexual-related disorders. This finding leads to the broader question of whether the Internet is a vector for already existing addictions or pathological conditions (gamblers using the Internet, certain paraphilia being more accessible on the Internet), or whether it is a primary cause of addiction (MMORPG addiction as a specific Internet issue).

Another interesting finding was the high percentage of people who have low PIU symptoms at the first evaluation, as measured by an IAT score of = 70 with or without a co-morbid diagnosis. One explanation is the lack of a clear diagnosis and the self-perception of this population. It is also possible that for people with an IAT score = 70, PIU has to be conceptualized as a secondary disorder. Another possibility is related to the debate over high versus low cut-off scores and the overall validity of Internet addiction scales. We chose the option of the high cut-off to limit false-positive diagnoses, as suggested in recent publications on PIU assessment tools. IAT is also a self-report questionnaire with its inherent limitations (social desirability, denial, shame, lack of introspection). These could be important biases, as IAT was self-administered during the first clinical interview.

All patients who consulted our clinic were supposed to have identified themselves as problematic Internet users. However, many of the patients were teenagers who were referred to the clinic by their families. The level of tolerance of the families for Internet usage and the individual perception of harmful usage may vary greatly. Scores on the IAT as low as 20 in our sample (i.e., answering “never” to all questions) might reflect either the patient’s denial of PIU or the exaggerated concerns of the patient’s relatives.

In particular for patients with no diagnosis, a brief intervention targeting psycho-education and reassurance, including a session with the relatives, is effective. For patients without PIU but who have a psychiatric diagnosis, the perception of PIU by the patient or the relatives might be a way to identify psychiatric symptoms early on, such as ADHD and social phobia in adolescents.

Concerning the mode of consultation, a parallel can be made with pathological gambling, where those who seek treatment are generally either under pressure (mostly from close relatives or under financial pressure) or experiencing secondary psychiatric symptoms such as anxiety and depression (Hodgins, Stea & Grant, 2011). The fact that PIU (and more specifically gaming) and gambling share similar elements might explain the relatively low overall consultation rate, high rate of co-morbid diagnosis at presentation, high rate of referral from family or caregivers compared with self-referral, and high dropout rate.

The patients who remained in treatment showed an overall improvement of symptoms. No patients worsened during treatment. The median length of treatment was 4 weeks and six sessions.

The study sample was small and the population heterogeneous in terms of motives for seeking assistance and co-morbid diagnosis. This was a case series based on clinical data obtained from patient charts. A prospective study with more detailed data collection could bring more accurate results in terms of psychiatric diagnosis (in our study, it was based on a semi-structured psychiatric interview at baseline) and better characterization of PIU. Since Internet addiction was not a formal diagnosis in our study, the IAT cut-off of 70 was chosen to diagnose PIU. IAT was measured only at evaluation, and the clinical evolution was measured by the CGI.

The overall positive evolution and the short mean duration of treatment may be clinically explained by the overall positive response to treatment. If PIU is a behavioral problem, because the patients are fairly young (mean age 24 years), we may not see a marked deterioration caused by the toxicity of the product as we do in substance abuse, and the duration and effect of the behavior may be short. These hypotheses need to be further tested.
